# Super-stable SnO_2_/MoS_2_ enhanced the electrocatalytic hydrogen evolution in acidic environments†

**DOI:** 10.1039/d2ra03627d

**Published:** 2022-08-17

**Authors:** Kun Huang, Lan Yang, Yihong Gao, Shikuo Li, Hui Zhang, Fangzhi Huang

**Affiliations:** School of Chemistry and Chemical Engineering, Anhui University Hefei Anhui 230601 P. R. China huangfangzhi@163.com; School of Materials Science and Engineering, Anhui University Hefei Anhui 230601 P. R. China lishikuo@ahu.edu.cn zhhui@ahu.edu.cn

## Abstract

For electrocatalytic hydrogen evolution in acidic environments, the stability of catalysts has always been a significant factor restricting development. Here, we prepared a superstable SnO_2_/MoS_2_ coupled nanosheet array on carbon cloth (CC@SnO_2_/MoS_2_), exhibiting an overpotential of 166 mV at a current density of 10 mA cm^−2^. According to the results of various tests and theoretical calculations, it is shown that the establishment of SnO_2_/MoS_2_ interface engineering is to accelerate the electron transmission on the heterogeneous interface and S defects on the edge of MoS_2_, and finally improve the conductivity and catalytic activity of the catalyst. More importantly, the formation of an SnO_2_ interface layer during *in situ* transformation improves the stability and hydrophilicity of the material surface. We have proposed a strategy for engineering an interface with fast electron transport and proton adsorption, providing some new ideas for the design of HER catalysts in acid electrolytes.

## Abstract 2

We designed a superstable SnO_2_/MoS_2_ coupled nanosheet array on carbon cloth, which exhibited interface engineering of SnO_2_/MoS_2_ with fast electron transfer and proton adsorption boosting electrocatalytic hydrogen evolution in acidic environments.

## Graphical abstract

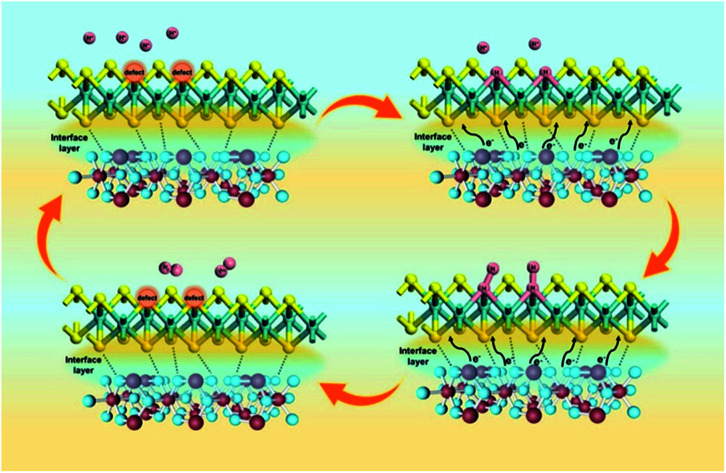

## Introduction

Benefiting from the advantages, a pollution-free and simple equipment, electrocatalytic hydrogen evolution technology is expected to replace the current industrial hydrogen production technology with high pollution and consumption.^1–5^ However, high-performance HER electrocatalysts expected to industrialization are precious metals such as Pt, which cannot acheive producation with large scale due to their rareness at present.^6,7^ Therefore, non-noble metal based electrocatalysts with high efficiency are urgently needed in the field of electrocatalytic hydrogen production.^8,9^ Compared with basic electrolytes, acidic electrolytes contain a large amount of free H^+^, which is more conducive to the occurrence of the electrocatalytic hydrogen evolution reaction.^10–12^ Unfortunately, the stability of most transition metal electrocatalysts in acidic solutions is so insufficient that the research of acidic HER catalysts is behind that of alkaline catalysts.^13,14^ Therefore, it is of practical value to develop non-noble metal electrocatalytic HER catalysts in acidic electrolytes.^15–17^ Some strategies have been developed to prepare acidic HER catalysts with low consumption or no precious metals.^18,19^ Liu *et al.* prepared a single-atom Pt catalyst on the surface of amorphous MoO_3_ and performed the HER in an acidic environment.^20^ The construction of monoatomic Pt contributes to the low consumption of precious metals and high performance, and the acid resistance of MoO_3_ can improve the corrosion resistance of the material.^21–23^ Using acid-resistant materials such as carbon materials to provide a protective layer for some transition metal semiconductors or materials is another promising strategy.^24–27^ However, instability and cumbersome problems in the preparation process will exist in the above strategies.^13,28^ Perhaps another way to think about it is that exploring materials with excellent intrinsic properties of acid resistance is an effective strategy to avoid the above triviality and instability.^29–33^

Molybdenum disulfide (MoS_2_) has excellent acid resistance, electrochemical properties and abundant reserves, and has been widely used in the field of electrocatalysis.^34,35^ Its catalytic activity is mainly derived from the unsaturated S atom edge of its layered structure, as shown in some reports.^36–40^ Therefore, increasing the edge part of MoS_2_ is an effective way to improve its catalytic performance. It has been reported that changing the morphology of molybdenum disulfide, such as nanoplatelets, nanoparticles, and quantum dots, expands the number of edges.^30,31,41^ Among them, dispersing MoS_2_ nanoparticles on an acid-resistant nanosheet array is a feasible method to increase the number of unsaturated S atoms and prevent the aggregation.^30^ Secondly, it is possible to construct the defects and vacancies of MoS_2_ to increase the degree of unsaturation at the edge of the S atom, thereby increasing the number of active sites. The research of Baker *et al.* showed that the competitive reaction between metal ions (such as Mo^4+^ and Sn^4+^) and non-metals in the *in situ* conversion process can effectively produce more defects.^42^ Finally, the resistance of electron transfer between the layers of MoS_2_ leads to poor conductivity and activity, which hinders the catalytic performance.^43,44^ The electron injection of the conductive medium into the molybdenum disulfide can effectively reduce this resistance.^30,45^ In summary, growing MoS_2_ nanoparticles on a nanosheet array with high conductivity and strong corrosion resistance through the competitive reaction of ions in the solution is a promising strategy. The thus obtained SnO_2_/MoS_2_ interface layer with fast electron transfer and proton adsorption is expected to be developed as a highly efficient and stable non-noble metal self-supporting HER electrocatalyst in acidic electrolytes.

Following the above idea, we used SnS_2_ grown on carbon cloth as a precursor, and MoS_2_ particles were grown on it to obtain a SnO_2_/MoS_2_ coupled nanosheet array. First, the uniform distribution of MoS_2_ nanoparticles on the nanosheets and the conversion of sulfides to oxides during the reaction increase the number of MoS_2_ unsaturated sulfur defects; secondly, the good hydrophilicity and stability of the SnO_2_ nanosheet substrate enhanced the hydrophilicity and stability of the catalyst;^46^ the heterogeneous interface layer formed by the coupling of SnO_2_ and MoS_2_ can accelerate the injection of electrons into MoS_2_ and reduce the resistance to electron transfer between molybdenum disulfide layers. The SnO_2_/MoS_2_ heterogeneous nanosheet arrays obtained by us maintain good activity and stability in acidic electrolytes. At a current density of 10 mA cm^−2^, the overpotential is 166 mV, which is significantly lower than that of pure MoS_2_ and SnO_2_. In the 0.5 M H_2_SO_4_ electrolyte, the catalyst can be utilized for more than 20 hours, accompanied by a current density decay within an acceptable range (less than 20%). Experimental results have proved that our proposed strategy of constructing an interface layer in designing an efficient and stable non-noble metal self-supporting HER electrocatalyst in an acidic electrolyte is effective and promising.

## Experimental

### Chemicals and materials

WOS1009 carbon cloth (CC) was purchased from Taiwan CeTech Co., Ltd. Other chemicals were of analytical grade and used as received without further purification. They were purchased from Shanghai Aladdin Biochemical Technology Co., Ltd and Sinopharm Chemical Reagent Co., Ltd. Ultrapure water was used throughout the experiments.

### Pretreatment of carbon cloth

The conductive carbon cloth was soaked in concentrated nitric acid and allowed to stand for three days. The soaked carbon cloth was rinsed three times with ultrapure water, and different solvents were used for ultrasonic cleaning for 10 minutes. The obtained carbon cloth was soaked in absolute ethanol for later use.

### Synthesis of CC@SnS_2_ NSs

The typical synthesis process of CC@SnS_2_ nanosheets is conducted by the solvothermal method. 140 mg of SnCl_4_·5H_2_O was dissolved in 20 mL of absolute ethanol, and then 60.4 mg of TAA (thioacetamide) was added and rotated for 20 min. The resulting liquid was injected into a 50 mL reactor. The treated carbon cloth was leaned against the bottom of the reaction kettle so that the carbon cloth was completely immersed in the solution, which was placed in an oven to react at 100 °C for 6 h, and then cooled naturally. The obtained sample was washed three times with water and C_2_H_5_OH each. The finally obtained CC@SnS_2_ nanosheets were activated in a vacuum drying oven at 70 °C for 12 h.

### Synthesis of CC@SnO_2_@MoS_2_ NSs

CC@SnO_2_@MoS_2_ nanosheets are synthesized by the hydrothermal method. 1 mL, 2 mL, 3 mL and 4 mL of 1.44 mg mL^−1^ TAA ethanol solution were injected into four clean beakers in turn. Then, 1 mL, 2 mL, 3 mL and 4 mL of 4.84 mg mL^−1^ molybdate solution were added, respectively. After mixing, the above solutions were diluted to 20 mL with ethanol respectively. The above solution was transferred to four 50 mL reactors, and the synthesized CC@SnS_2_ nanosheets were leaned against the bottom of the reactor to completely immerse the carbon cloth in the solution. The reaction was carried out at 200 °C for 12 h. After cooling down, the obtained sample was washed with water and C_2_H_5_OH three times each. The finally obtained CC@SnO_2_@MoS_2_ nanosheets were dried in a vacuum drying oven at 70 °C for 12 h. The different samples obtained were named CC@SnO_2_@MoS_2_-1, CC@SnO_2_@MoS_2_-2.5, CC@SnO_2_@MoS_2_-5, and CC@SnO_2_@MoS_2_-10.

### Synthesis of CC@MoS_2_ NSs

The synthesis of CC@MoS_2_ nanosheets is roughly the same as the above CC@SnO_2_@MoS_2_-5 nanosheets except just replacing CC@SnS_2_ with conductive carbon cloth.

### Synthesis of CC@SnO_2_ NSs

The solvothermal method was used to prepare CC@SnO_2_ NSs. 140 mg of SnCl_4_ 5H_2_O and the corresponding Na_3_C_6_H_5_O_7_ 2H_2_O were dissolved in 20 mL of distilled water. After stirring the solution for 5 minutes, a certain concentration of NaOH solution was added. The resulting liquid was injected into a 50 mL reactor. The treated carbon cloth was leaned against the bottom of the reaction kettle so that the carbon cloth was completely immersed in the solution, which was placed in an oven to react at 180 °C for 12 h, and then cooled naturally. The obtained sample was washed three times with water and C_2_H_5_OH each. The finally obtained CC@SnO_2_ nanosheets were activated in a vacuum drying oven at 70 °C for 12 h.

## Results and discussion

**Scheme 1:**
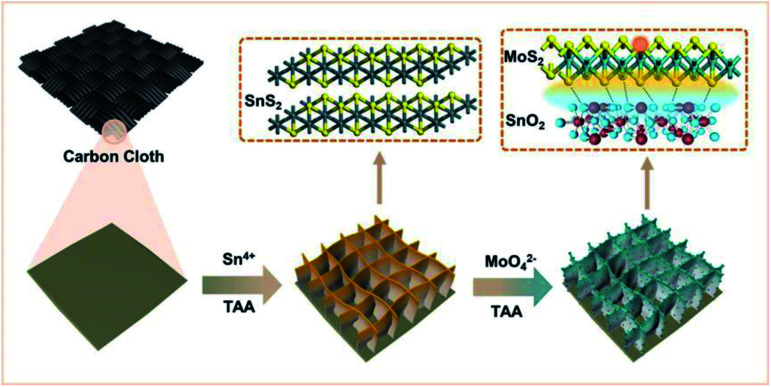
Diagram of material synthesis.

**Fig. 1:**
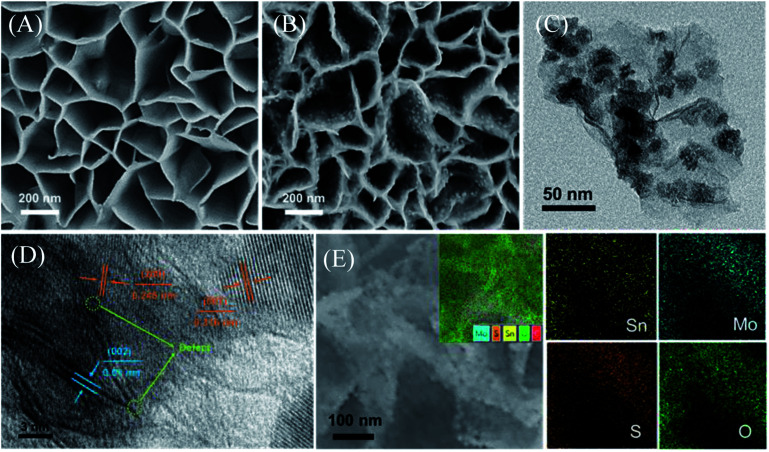
(A) SEM image of CC@SnS_2_ NSs. (B) SEM image of CC@SnO_2_/MoS_2_ NSs. (C) TEM images of SnO_2_/MoS_2_ NSs. (D) HRTEM images of SnO_2_/MoS_2_ NSs. (E) Element distribution mapping of Sn, Mo, S and O corresponding to SnO_2_/MoS_2_ NSs.

A schematic diagram of the material synthesis is shown in Scheme 1. First, SnS_2_ NSs are constructed on the carbon cloth substrate using the solvothermal method. The thickness of the SnS_2_ nanosheets is about 10 nm in the SEM image (Fig. 1A), growing uniformly and vertically on the carbon cloth.

The honeycomb-like porous structure of vertical array is open-type so that it is more conducive to the introduction of electrolytes. Subsequently, MoS_2_ nanoparticles with uniform distribution and very uniform diameter were grown on the surface of SnS_2_, as shown in Fig. 1B. SnS_2_ will also be converted to SnO_2_ at this time, which is confirmed in the following characterization analysis. In addition, some MoS_2_ nanosheets will epitaxially grow along the edge of the SnO_2_ nanosheets, which also exposes more MoS_2_ unsaturated S edges. With the increase of S source and Mo source, MoS_2_ on the surface of SnO_2_ gradually transformed into nanoparticles, and finally formed multiple folds covering the surface, shown in the SEM of samples (Fig. S1A–D†). The SEM results indicate that the coverage of the folds from nanoparticles to MoS_2_ on the substrate is controllable. The combination of MoS_2_ nanoparticles and SnO_2_ nanosheets can be clearly observed from the transmission electron microscope image (TEM), as shown in Fig. 1C. The uniformly distributed MoS_2_ nanoparticles shown in the image will not agglomerate and have a diameter of about 25 nm. The high-magnification TEM of SnO_2_/MoS_2_ NSs clearly shows the lattice spacing of SnO_2_ and MoS_2_, as shown in Fig. 1D. The lattice spacing of SnO_2_ is 0.245 nm and 0.316 nm, and the distribution corresponds to the (200) and (001) crystal planes. In addition, the crystal lattice fracture is clearly observed, which corresponds to the defects formed during the crystal phase transformation. The formation of these defects is closely related to the competitive reaction of non-metal elements in the solvothermal reaction process. The lattice spacing of 0.61 nm corresponds to the (002) crystal plane of MoS_2_. The element distribution image of SnO_2_/MoS_2_ NSs shows that Sn, Mo, S, and O are evenly distributed on the nanosheet, which reveals the existence and distribution of each element, confirming the accomplished loading of MoS_2_ (Fig. 1E).

**Fig. 2:**
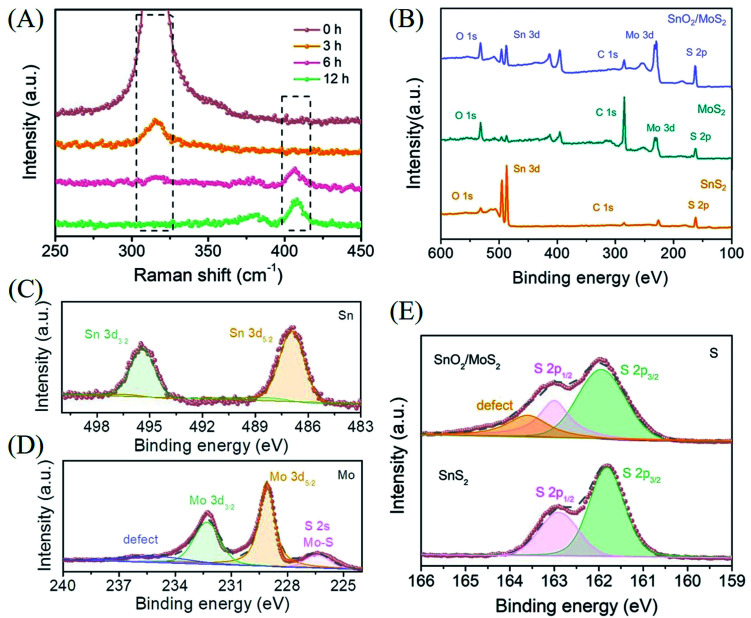
(A) Raman image of different reaction times during the growth of MoS_2_. (B) XPS general map of SnO_2_/MoS_2_ NSs. High resolution XPS image of SnO_2_/MoS_2_ NSs: (C) Sn in SnO_2_/MoS_2_ NSs, (D) Mo in SnO_2_/MoS_2_ NSs, and (E) S in SnO_2_/MoS_2_ NSs and SnS_2_ NSs.

The results of XRD prove that the phase change from sulfide to oxide actually occurred during the second step of the reaction (Fig. S2A†). However, due to the low content and poor crystallinity of MoS_2_ on the surface as well as the strong peak of SnO_2_, the corresponding diffraction peak of MoS_2_ is difficult to find. In order to completely prove that the samples are loaded with MoS_2_ and to research the details of the transition from SnS_2_ to SnO_2_, we performed Raman spectroscopy analysis with different reaction times (Fig. 2A). In the unreacted pure SnS_2_ curve, a single strong Raman peak is observed at 317 cm^−1^, which corresponds to the A_1g_ characteristic peak of SnS_2_. When the reaction time is 3 h, the intensity of the characteristic peak corresponding to 317 cm^−1^ is significantly reduced, indicating that the SnS_2_ composition is decreasing. Since Sn ions are more easily coordinated with oxygen in solution and are less sulfur-loving than Mo ions, the transformation of SnS_2_ to SnO_2_ has begun. However, the characteristic peak of MoS_2_ is still not observed at this time, which reflects that the formation of MoS_2_ requires more time or is later than the transformation of SnS_2_. We prefer the former. As the formation reaction of MoS_2_ has begun, the more sulfur-philic nature of Mo compared to Sn promotes the transition from SnS_2_ to SnO_2_ to occur more quickly. Accompanied by a reaction time of 6 h, the disappeared A_1g_ characteristic peak of SnS_2_ indicates that the phase change from SnS_2_ to SnO_2_ has been completely completed. The E_12g_ in-plane and A_1g_ out-of-plane characteristic peaks corresponding to MoS_2_ at 380 and 408 cm^−1^ indicate that MoS_2_ has initially formed. With the reaction for 12 hours, the further enhanced characteristic peaks at 380 and 408 cm^−1^ corresponded to the complete transition from SnS_2_ to SnO_2_/MoS_2_. The results of the Raman diagram indicate that the competition between Sn and Mo for S^2−^ does exist during the reaction, which leads to the transformation of Sn from sulfide to oxide.

In order to further research the valence composition of typical SnO_2_/MoS_2_ nanosheets, we performed XPS characterization of the samples. The characteristic peaks of Mo and Sn further proved the successful preparation of SnO_2_/MoS_2_ nanosheets, shown in the XPS total spectrum (Fig. 2B). The high-resolution XPS spectra of each element in SnO_2_/MoS_2_ NSs have been obtained (Fig. 2C–E). Sn 3d_5/2_ and Sn 3d_3/2_ located at 486.9 eV and 495.4 eV, respectively, are attributed to the characteristic peaks of Sn^4+^, indicating the presence of positive tetravalent Sn elements in the material (Fig. 2C). The formation of MoS_2_ is also certified by two characteristic peaks in the high-resolution XPS spectrum of Mo, as shown in Fig. 2D. The characteristic peaks at 229.1 eV and 232.3 eV correspond to Mo 3d_5/2_ and Mo 3d_3/2_, which confirms the above point. The S 2s energy peak near 226.4 eV is derived from the Mo–S bond, which once again verifies the existence of molybdenum disulfide. A broad peak can also be observed at 235.4 eV, which may be related to the defects in the competitive reaction. The characteristic peaks of S 2p_2/3_ and S 2p_1/3_ are observed at 161.96 eV and 163.0 eV, indicating that the valence states of S elements in SnS_2_ and SnO_2_/MoS_2_ are all negative divalent (Fig. 2E). And a peak can be observed at 163.6 eV, which is attributed to the defects of the unsaturated sulfur atoms contained. The XPS results once again confirmed that we successfully synthesized the SnO_2_/MoS_2_ nanosheet structure, and the sample contains a large number of defects that can provide active sites due to the competitive reaction of metal ions for S^2−^.

**Fig. 3:**
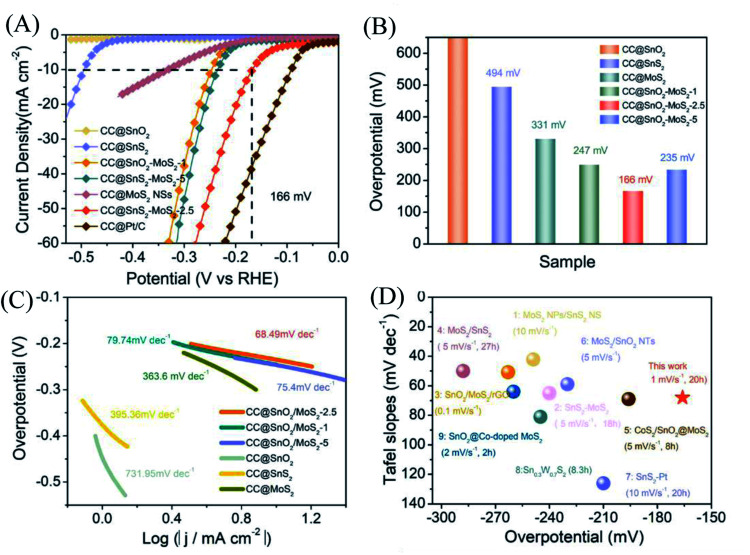
(A) LSV curves of different SnO_2_/MoS_2_ NSs (1, 2.5, and 5), SnS_2_, SnO_2_, Pt/C and MoS_2_ at 1 mV s^−1^. (B) Overpotential (*η*) of different samples at 10 mA cm^−2^. (C) Tafel slope plots corresponding to different samples. (D) A performance comparison diagram of this work with the work in 0.5 M H_2_SO_4_ of published relevant materials, with the sweep rate of the LSV curve and the stabilization time at 10 mA cm^−2^ in parentheses.^29–32,47–52^

With a scan rate of 1 mV s^−1^, CC@SnO_2_/MoS_2_ NS samples were tested for HER performance in 0.5 M H_2_SO_4_ electrolyte. The comparative samples are CC@Pt/C, CC@SnS_2_, CC@SnO_2_ and CC@MoS_2_ with roughly the same load mass. All electrocatalytic results have been compensated for resistance to obtain accurate catalyst intrinsic performance. As shown in Fig. 3A, the CC@SnO_2_/MoS_2_ NS-2.5 sample exhibits a current density of 10 mA cm^−2^ at an overpotential of only 166 mV, which is better than other comparative samples. Pt/C exhibits the best activity as a conventional HER catalyst with an overpotential of 90 mV. It is worth noting that the overpotential of CC@SnO_2_/MoS_2_ NSs-2.5 is the lowest, better than samples with other loadings (Fig. 3B). This means that only the most suitable load of MoS_2_ can maximize performance. The CC@SnO_2_ sample has poor electrocatalytic activity, which makes it difficult to measure the standard current density. Compared with CC@SnS_2_ (494 mV) and CC@MoS_2_ (330 mV), the overpotential of CC@SnO_2_/MoS_2_ NSs-2.5 has a voltage reduction of 330 mV and 164 mV, respectively. The Tafel slope of CC@SnO_2_/MoS_2_ NSs-2.5 is 68.49 mV dec^−1^, which is much smaller than that of the other comparative samples, indicating that the CC@SnO_2_/MoS_2_ NS-2.5 sample has the most excellent electrochemical reaction kinetics among many materials (Fig. 3B). The results of LSV and Tafel slope show that we optimize electron transfer, proton adsorption and wettability of MoS_2_ through designing the SnO_2_/MoS_2_ interface layer, which is reflected in the catalytic performance.

**Fig. 4:**
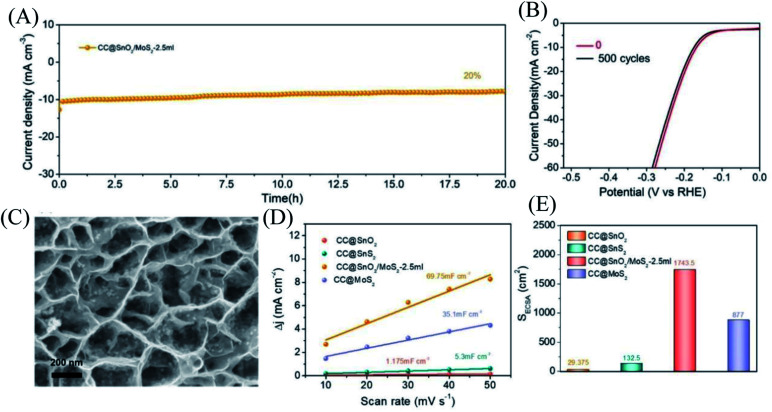
(A) Curve of *i*–*t* with a constant current density of 10 mA cm^−2^. (B) LSV curve comparison of the SnO_2_/MoS_2_ NS-2.5 sample in 0 cycle and 500 cycles. (C) SEM image of SnO_2_/MoS_2_ NS-2.5 sample after 500 cycles. (D) The curve between CV scanning rate and current density of each sample. (E) The electrochemical active area of each sample.

**Fig. 5:**
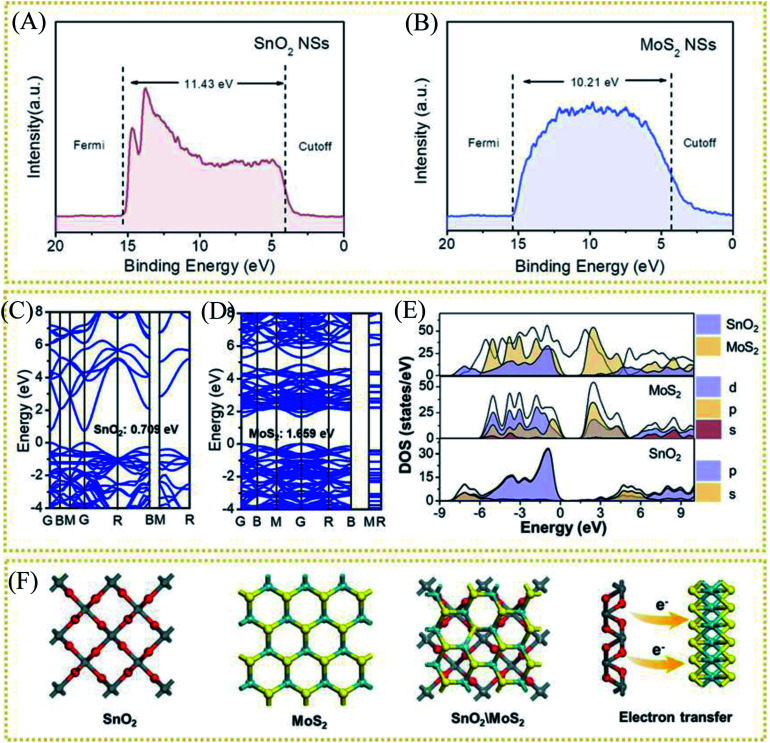
Ultraviolet photoelectron spectroscopy (UPS) of (A) CC@SnO_2_ NSs and (B) CC@SnS_2_ NSs. (C) Calculation of band structure of SnO_2_ and (D) MoS_2_. (E) DOS and PDOS results for SnO_2_, MoS_2_ and SnO_2_/MoS_2_. (F) Modeling results of SnO_2_, MoS_2_ and SnO_2_/MoS_2_.

For electrocatalysts used in acid electrolytes, the stability is a significant parameter for evaluating catalysts. The *i*–*t* curve of the CC@SnO_2_/MoS_2_ sample was recorded with a voltage of 166 mV in order to test the stability of performance (Fig. 5A). After 20 hours of continuous electrolysis, samples with less than 20% attenuation of current indicate that the synthesized CC@SnO_2_/MoS_2_ heterogeneous nanosheets are expected to achieve long-term electrocatalytic processes. The test of the continuous 500-cycle cyclic voltammetry curve without obvious attenuation shows that the catalyst can withstand continuous catalytic reactions of different potentials and shows good stability (Fig. 4B). The sample after 500 cycles maintained the original morphology and showed strong toughness (Fig. 4C). The performance of this work is compared with the reported performance of related materials 1–9, as shown in Fig. 3D.^29–32,47–52^ The abscissa and ordinate of the graph are the overpotential of the catalyst at 10 mA cm^−2^ and the Tafel slope, respectively. The overpotential of the electrode material reported in this work is better than that of related materials, and its Tafel slope reflects that the electrochemical kinetics also has outstanding competitiveness. The electrochemical active area is usually an important basis for evaluating the intrinsic activity of a catalyst. The double-layer capacitor area of the material can be used to estimate the size of the electrochemically active area, as shown in Fig. S3.† The linear function image of the CV scan rate and current density difference (Δ*j*) is obtained by fitting (Fig. 4E). From the figure, we can acquire that the *C*_dl_ of samples CC@SnO_2_ NSs, CC@SnS_2_ NSs, CC@MoS_2_ NSs and CC@SnO_2_/MoS_2_ NSs-2.5 is 1.175, 5.3, 35.1 and 69.75 mF cm^−2^, respectively. The electrochemically active area of the CC@SnO_2_/MoS_2_ NS-2.5 sample is 1743.5 cm^2^, which is much higher than that of CC@MoS_2_ NSs and other samples (Fig. 4F). On the basis of the above results, we also calculated ESCA-normalized LSV curves to confirm the intrinsic activity of the catalysts (Fig. S4†). With the largest electrochemical active area, SnO_2_@MoS_2_ still has the best intrinsic activity compared to other samples. The measurement results of electrochemically active area show that the construction of an interface layer in CC@SnO_2_/MoS_2_ nanosheets can effectively increase the number of active sites.

Furthermore, the hydrophilicity of electrode materials affects the performance of electrocatalytic hydrogen production. Hydrophilic samples can absorb more protons, and the H_2_ generated on the surface is easier to desorb, which is more conducive to the occurrence of the HER. The surface wettability of CC@MoS_2_ NS and CC@SnO_2_/MoS_2_ NS-2.5 samples was compared (Fig. S5A and B†). The surface of the CC@MoS_2_ sample is hydrophobic and has weak adhesion to liquids. The CC@SnO_2_/MoS_2_ NS-2.5 sample turned into a surface hydrophilic material, which may be the result of the improvement of the overall material due to the hydrophilic nature of SnO_2_. In addition, the electrical conductivity of the material has also been studied through the electrochemical impedance test, as shown in Fig. S6.† The electrochemical impedance test shows that the impedance of the SnO_2_ nanosheet structure grown with MoS_2_ nanoparticles is much smaller than that of pure MoS_2_ and pure SnO_2_ nanosheets, indicating that the construction of the SnO_2_/MoS_2_ interface layer speeds up electron transmission and reduces the resistance of the material. All these are further illustrated in the resistance fitting curve and equivalent circuit of the electrochemical impedance spectrum of SnO_2_/MoS_2_ (Fig. S7†).

As mentioned above, through the electron injection of SnO_2_ into MoS_2_, the design of the interface layer reduces the resistance of the lateral surface of MoS_2_ to electron transfer and the strong van der Waals effect between layers to improve the conductivity. To study the electron transfer relationship between SnO_2_ and MoS_2_, the UV photoelectron spectroscopy (UPS) test of pure MoS_2_ and pure SnO_2_ was used to calculate their respective work functions (*Φ*). When an electron transitions from the inside of the conductor to the outside of the conductor, the minimum energy value required is *Φ*. Therefore, electrons will be transferred from components with a small work function to components with a large work function, accelerating electron transport. The UV photoelectron spectroscopy shows the value of the Fermi edge (*E*_Fermi_) and the truncated edge (*E*_cut off_) of the sample, as shown in the formula.*Φ* = *hυ* + *E*_cut off_ − *E*_Fermi_

**Fig. 6:**
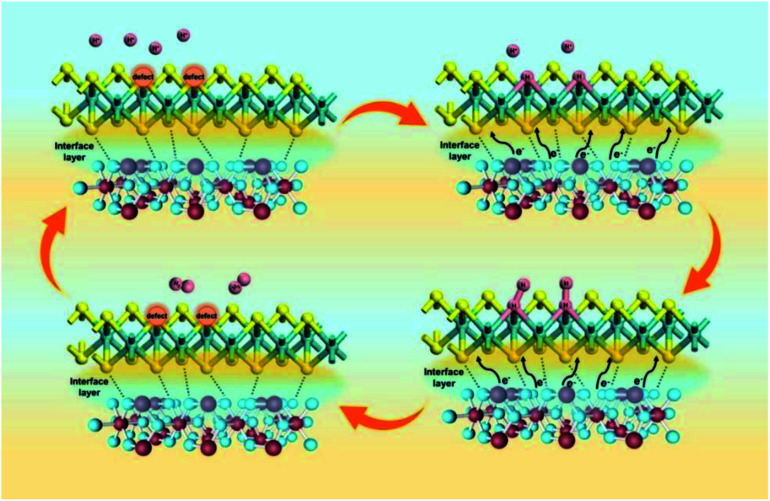
Electron transfer simulation diagram of the SnO_2_/MoS_2_ NS heterogeneous interface layer.

Among them, *Φ* is the work function of the material, also known as work function; *hυ* is the monochromatic light source with a value of 21.2 eV. The UPS energy spectra of pure MoS_2_ and pure SnO_2_ are shown in Fig. 5A and B. The difference value of SnO_2_ between the Fermi edge and the truncated edge is 11.43 eV (Fig. 5A). Similarly, for MoS_2_, the calculation result of the difference value between the Fermi edge and the truncated edge is 10.21 eV (Fig. 5B). According to the formula, the work functions of SnO_2_ NSs and MoS_2_ NSs are 9.79 eV and 11.01 eV, respectively. The result shows that less energy is required for electrons in SnO_2_ to escape from the inside of the material to the surface of the material, which confirms the electron injection of SnO_2_ into MoS_2_ during the catalysis process. The previous performance test also demonstrates that this electron transfer does enhance the performance of the catalyst. The energy band structure and density of states (DOS) of SnO_2_, MoS_2_, and SnO_2_/MoS_2_ are also calculated to support the UPS test results, as shown in Fig. 5C and D. The theoretical calculation results of the energy band structure of SnO_2_ and MoS_2_ show that the band gap of SnO_2_ is only 0.709 eV, showing excellent conductivity (Fig. 5C). In the conduction band position, the p orbit and s orbit of SnO_2_ are more upward than those of MoS_2_, which means that the electrons of SnO_2_ are more easily transferred to the MoS_2_ component, which is consistent with the results of UPS (Fig. 5D). Fig. 5E is the simulation diagram of SnO_2_, MoS_2_ and SnO_2_/MoS_2_ corresponding to the above theoretical calculation respectively. A simulation diagram of the electron transfer of the SnO_2_/MoS_2_ NS heterogeneous interface layer is shown in Fig. 6. Due to the small work function of SnO_2_, electrons can be injected into the MoS_2_ component at an accelerated rate, which increases the electron transfer rate between the interface; on the other hand, due to the large amount of competing reactions in the reaction liquid unsaturated S atom defects, the structure of nanosheets/nanoparticles and the introduction of the SnO_2_ increase the number of active sites and improve the hydrophilicity of the material, making H^+^ easier to be adsorbed and reduced and H_2_ desorbed.

## Conclusions

To sum up, in order to construct an efficient and stable acidic HER electrocatalyst, using SnS_2_ nanosheets on carbon cloth as the precursor, we transformed SnS_2_ into SnO_2_ with hydrophilia and corrosion resistance through the competitive reaction for S^2−^ between Mo^4+^ and Sn^4+^. At the same time, MoS_2_ nanoparticles were grown on SnO_2_ nanosheets to obtain a SnO_2_/MoS_2_ interface layer with fast electron transfer and proton adsorption. The result of high-magnification TEM and XPS proved that the nanosheet/nanoparticle structure and the competitive reaction in the reaction solution increased the number of MoS_2_ unsaturated sulfur defects and effectively prevented their aggregation; secondly, the interface contact angle test shows that the introduction of SnO_2_ improved the overall hydrophilicity of the material, being beneficial to the HER; finally, the interface layer formed by SnO_2_ and MoS_2_ can accelerate the injection of electrons into molybdenum disulfide and reduce the resistance to electron transfer between molybdenum disulfide layers, which is proved by UPS and theoretical calculation. The typical SnO_2_/MoS_2_ heterogeneous nanosheets maintain good activity and stability in acid electrolytes. At a current density of 10 mA cm^−2^, the overpotential of the SnO_2_/MoS_2_ is only 166 mV, having a significant performance improvement, compared with pure MoS_2_, pure SnO_2_ and pure SnS_2_. We have proposed a strategy for constructing an interface layer with fast electron transport and proton adsorption, providing some new ideas for the design of HER catalysts in acid electrolytes.

## Author contributions

Kun Huang: conceptualization, investigation, formal analysis, data curation, methodology, writing – original draft, and writing – review & editing. Lan Yang: conceptualization, investigation, formal analysis, data curation, and validation. Yihong Gao: theoretical calculation. Hui Zhang: methodology and investigation. Shikuo Li: methodology and investigation. Fangzhi Huang: conceptualization, data curation, formal analysis, funding acquisition, resources, and supervision.

## Conflicts of interest

There are no conflicts to declare.

## Supplementary Material

RA-012-D2RA03627D-s001
