# Supplementary material for: Super-stable SnO2/MoS2 enhanced the electrocatalytic hydrogen evolution in acidic environments
Source: RSC Adv. 2022 Aug 17;12(36):23503–12. doi: 10.1039/d2ra03627d (PMC9382654; doi:10.1039/d2ra03627d)
Supplement: RA-012-D2RA03627D-s001 [file RA-012-D2RA03627D-s001.pdf]

Electronic Supplementary Material (ESI) for RSC Advances.
This journal is © The Royal Society of Chemistry 2022

Supplementary materials

Super-stable SnO_2/MoS_2 enhanced the electrocatalytic hydrogen evolution in acidic environment

Kun Huang, △[a] Lan Yang △[a], Yihong Gao[a], Shikuo Li*[b], Hui Zhang*[b],and Fangzhi Huang *[a]

Sample Characterizations:

Scanning electron microscopy (SEM) images were acquired on a Sigma 500 SEM instrument (SEISS, Germany). HRTEM images were performed on a JEM-2100 high-resolution transmission electron microscope. X-ray photoelectron spectroscopy (XPS)(ESCA Lab MKII) was used to detect the chemical composition of the sample. A powder X-ray diffraction (XRD) pattern was obtained using a Philips X'Pert PRO SUPER X-ray diffractometer equipped with graphite-monochromated Cu Kα radiation (λ= 1.54056 Å). The Raman spectra were collected using a Lab RAM HR800 confocal microscope Raman system (Horiba Jobin Yvon，632.8nm) with a 50× objective lens.

Electrochemical test

The electrochemical workstation used in this work was purchased from Shanghai Chenhua. The electrode combination mode is a three-electrode mode. The load of SnO_2/MoS_2 is about 0.035 g. It is used as the working electrode, the Platinum electrode is used as the counter electrode, and the 0.5 M H_2SO_4 aqueous solution is used as the electrolysis environment. The HER performance of CC@MoS_2, CC@SnO_2, and CC@SnS_2 catalysts with the same loading capacity were also measured for comparison, and the Ag/AgCl electrode was used as a reference electrode. When the scanning speed is 1 mV s^{-1}, the polarization curve is measured. The Tafel equation (η=a + b log j) can be used to find the Tafel slope from the LSV curve. Where η is the overpotential, a is the Tafel constant, b is the Tafel slope, and j is the current density. At the same time, a chronoamperometric test was performed to study the long-term stability of the catalyst at an overpotential of 166 mV. In the 0.2~0.4 V (vs. RHE) potential range (Faraday potential zone), the electric current is measured by measuring the double-layer capacitance (C_{dl}) at different scan rates of 10, 20, 30, 40, 50 mV s^{-1} Chemically active area (ECSA). The linear slope of scanning speed and Δj is twice that of C_{dl}. 95% resistance compensation is used to obtain the true catalytic efficiency.

Theoretical calculation model

The theoretical simulation calculation methods are all carried out using the academic version of CASTEP. The Perdew-Burke Ernzerhof (PBE) function based on Generalized Gradient Approximation (GGA) is used to process non-local exchange and

correlation energy. For the MoS_2 unit cell, the lattice parameters α=β=γ=90°, a=5.446 Å, b=3.15 Å, and c=12.3 Å. For the SnO_2 unit cell, the lattice parameters α=β=γ=90°, a = b = 4.73727Å, and c = 3.18Å. The SnO_2/MoS_2 interface is constructed with 2 × 3 units of MoS_2 (001) and 2 × 2 units of SnO_2 (001) surfaces.

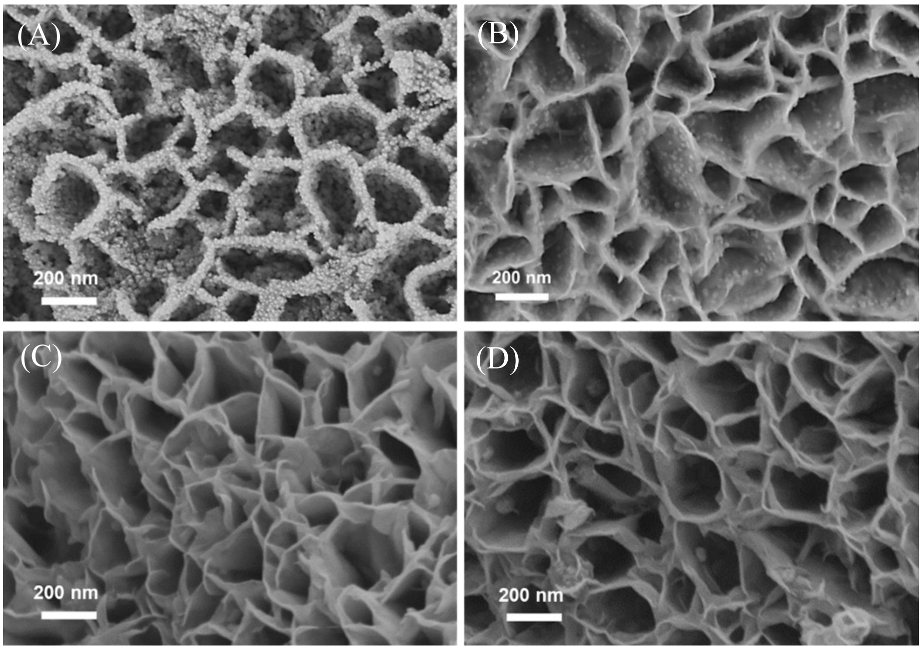

Fig. S1 SEM diagram of samples grown with different amounts of MoS_2 (A) CC@SnO_2/MoS_2 NSs-1 (B) CC@SnO_2/MoS_2 NSs-2.5 (C) CC@SnO_2/MoS_2 NSs-5 (D) CC@SnO_2/MoS_2 NSs-10.

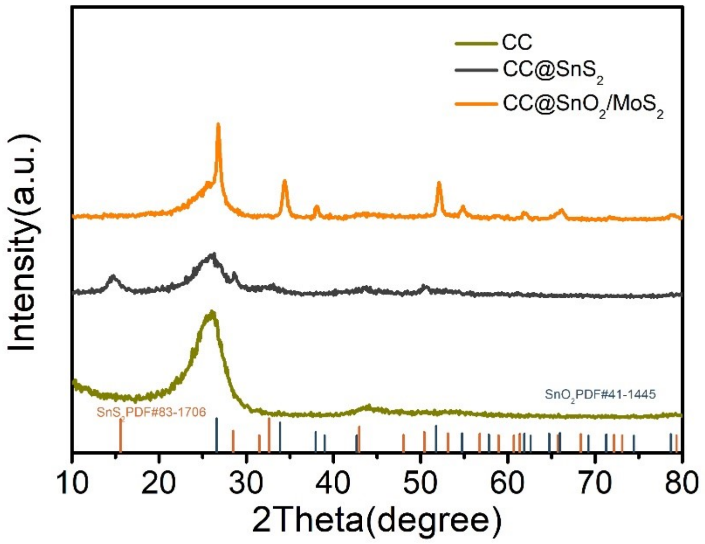

Fig. S2 X-ray Diffraction (XRD) patterns of SnO_2/MoS_2 NSs.

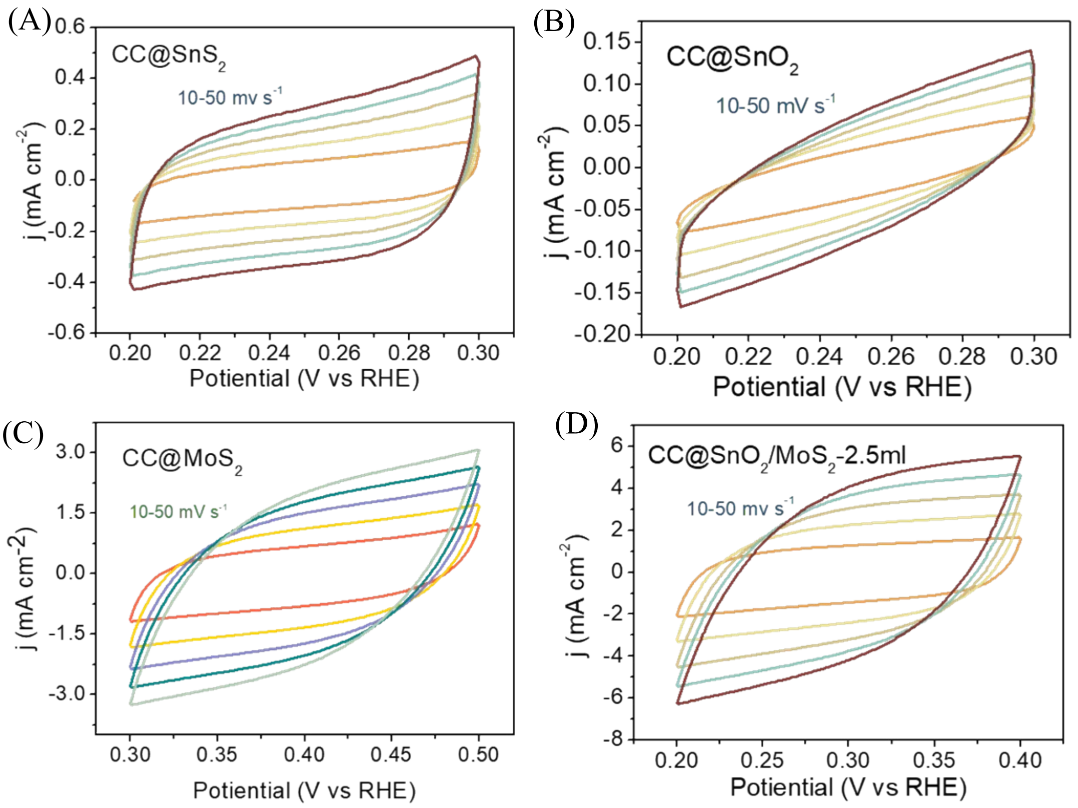

Fig. S3 CV of (A) CC@SnS_2 NSs (B) CC@SnO_2 NSs (C) CC@ MoS_2 NSs and (D) CC@SnO_2/MoS_2 NSs-2.5 at certain potential window at different scanning rates.

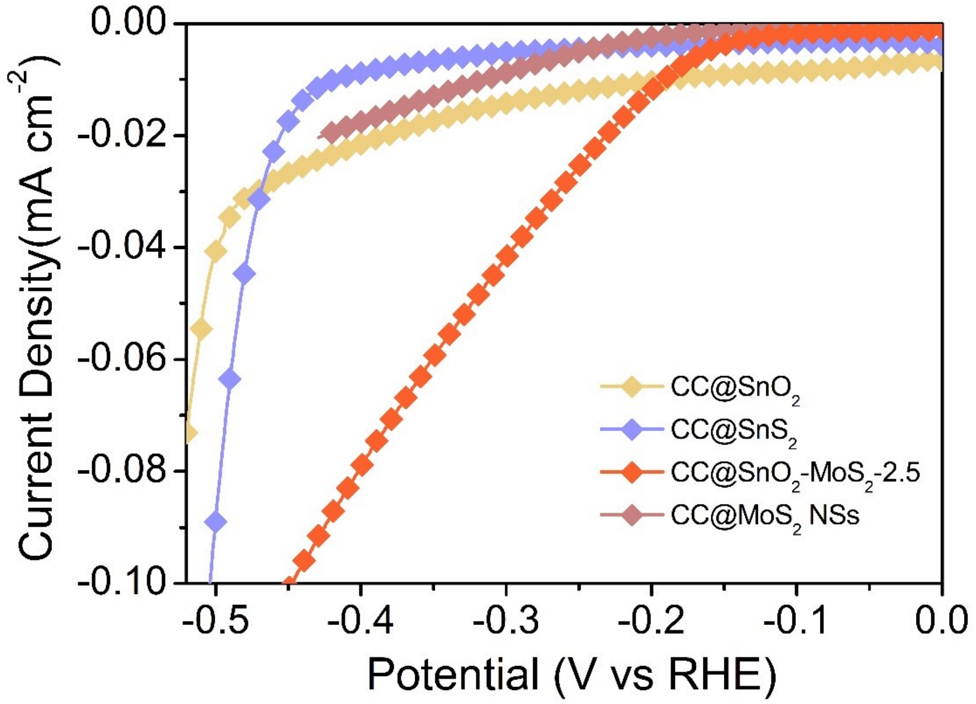

Fig. S4 LSV curves of different samples at 1 mV s^{-1} normalized by ECSA.

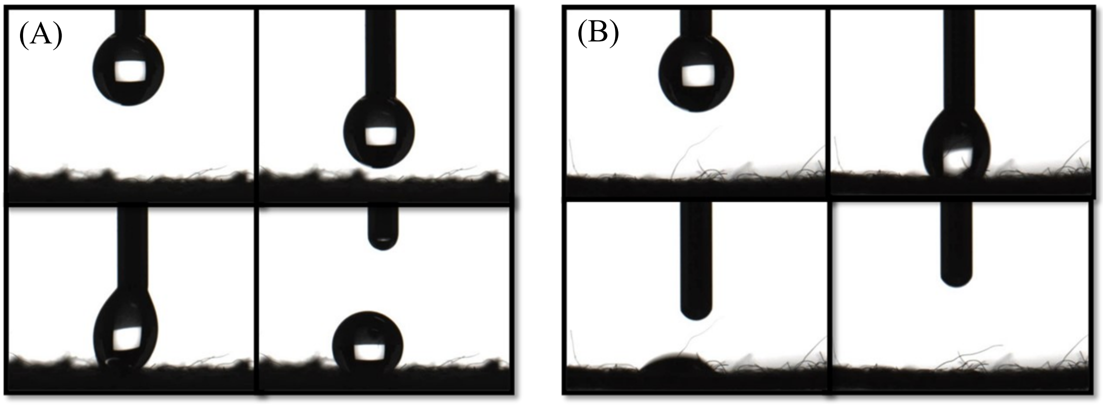

Fig. S5 Optical photos of the wettability of (A) CC@MoS_2 NSs and (B) CC@SnO_2/MoS_2 NSs-2.5 samples.

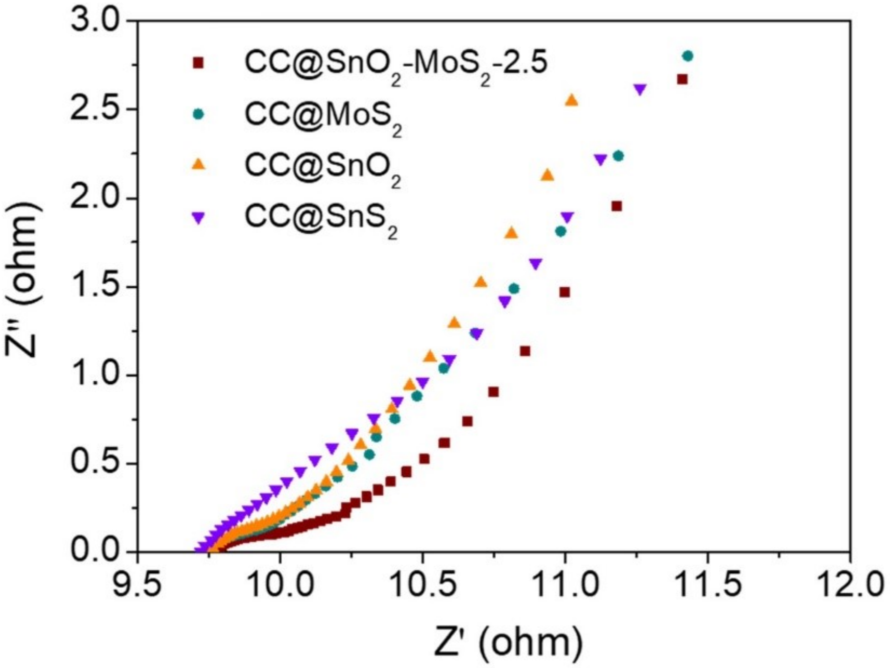

Fig. S6 Electrochemical Impedance (EIS) of CC@SnO_2 NSs, CC@SnS_2 NSs, CC@ MoS_2 NSs and CC@SnO_2/MoS_2 NSs-2.5.

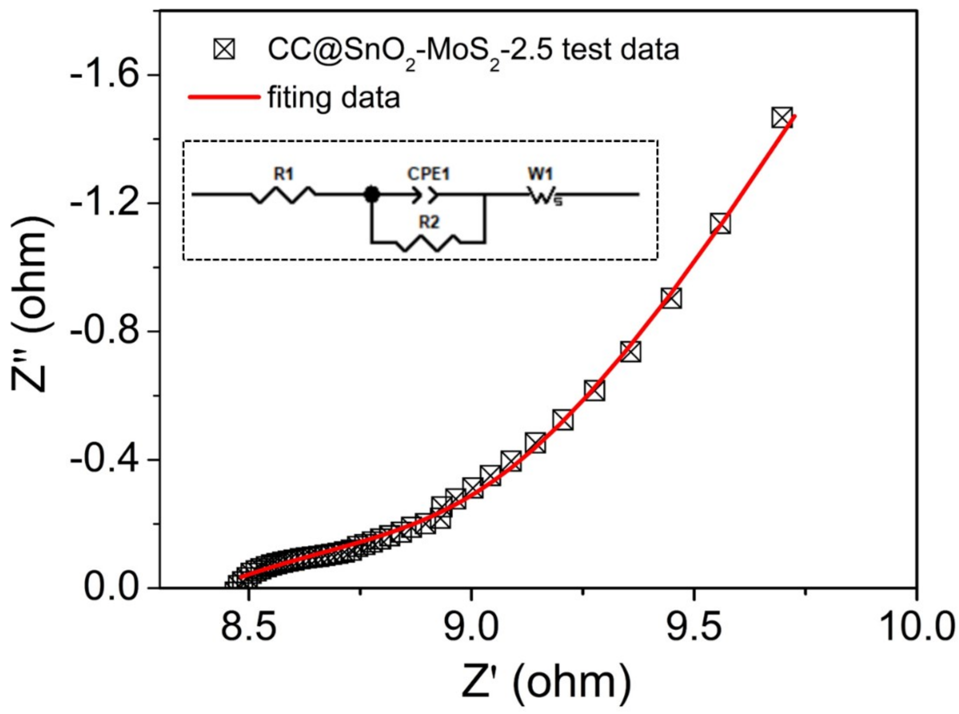

Fig. S7 The resistance fitting curve and equivalent circuit of electrochemical impedance spectra (ESI) of CC@SnO_2/MoS_2 - 2.5.
